# Role of the E2 Hypervariable Region (HVR1) in the Immunogenicity of a Recombinant Hepatitis C Virus Vaccine

**DOI:** 10.1128/JVI.02141-17

**Published:** 2018-05-14

**Authors:** John L. M. Law, Michael Logan, Jason Wong, Juthika Kundu, Darren Hockman, Amir Landi, Chao Chen, Kevin Crawford, Mark Wininger, Janelle Johnson, Catalina Mesa Prince, Elzbieta Dudek, Ninad Mehta, D. Lorne Tyrrell, Michael Houghton

**Affiliations:** a Li Ka Shing Institute of Virology, Department of Medical Microbiology and Immunology, University of Alberta, Edmonton, Alberta, Canada; University of Southern California

**Keywords:** glycoprotein, HCV, HVR1, neutralization, neutralizing antibody, RNA virus, vaccine, hepatitis C virus

## Abstract

Current evidence supports a protective role for virus-neutralizing antibodies in immunity against hepatitis C virus (HCV) infection. Many cross-neutralizing monoclonal antibodies have been identified. These antibodies have been shown to provide protection or to clear infection in animal models. Previous clinical trials have shown that a gpE1/gpE2 vaccine can induce antibodies that neutralize the *in vitro* infectivity of all the major cell culture-derived HCV (HCVcc) genotypes around the world. However, cross-neutralization appeared to favor certain genotypes, with significant but lower neutralization against others. HCV may employ epitope masking to avoid antibody-mediated neutralization. Hypervariable region 1 (HVR1) at the amino terminus of glycoprotein E2 has been shown to restrict access to many neutralizing antibodies. Consistent with this, other groups have reported that recombinant viruses lacking HVR1 are hypersensitive to neutralization. It has been proposed that gpE1/gpE2 lacking this domain could be a better vaccine antigen to induce broadly neutralizing antibodies. In this study, we examined the immunogenicity of recombinant gpE1/gpE2 lacking HVR1 (ΔHVR1). Our results indicate that wild-type (WT) and ΔHVR1 gpE1/gpE2 antigens induced antibodies targeting many well-characterized cross-genotype-neutralizing epitopes. However, while the WT gpE1/gpE2 vaccine can induce cross-genotype protection against various genotypes of HCVcc and/or HCV-pseudotyped virus (HCVpp), antisera from ΔHVR1 gpE1/gpE2-immunized animals exhibited either reduced homologous neutralization activity compared to that of the WT or heterologous neutralization activity similar to that of the WT. These data suggest that ΔHVR1 gpE1/gpE2 is not a superior vaccine antigen. Based on previously reported chimpanzee protection data using WT gpE1/gpE2 and our current findings, we are preparing a combination vaccine including wild-type recombinant gpE1/gpE2 for clinical testing in the future.

**IMPORTANCE** An HCV vaccine is an unmet medical need. Current evidence suggests that neutralizing antibodies play an important role in virus clearance, along with cellular immune responses. Previous clinical data showed that gpE1/gpE2 can effectively induce cross-neutralizing antibodies, although they favor certain genotypes. HCV employs HVR1 within gpE2 to evade host immune control. It has been hypothesized that the removal of this domain would improve the production of cross-neutralizing antibodies. In this study, we compared the immunogenicities of WT and ΔHVR1 gpE1/gpE2 antigens as vaccine candidates. Our results indicate that the ΔHVR1 gpE1/gpE2 antigen confers no advantages in the neutralization of HCV compared with the WT antigen. Previously, we showed that this WT antigen remains the only vaccine candidate to protect chimpanzees from chronic infection, contains multiple cross-neutralizing epitopes, and is well tolerated and immunogenic in humans. The current data support the further clinical development of this vaccine antigen component.

## INTRODUCTION

Hepatitis C virus (HCV) is a major global health concern infecting between 70 million and 150 million people worldwide (1, 2). A successful direct antiviral treatment is now available to cure most patients. However, the high cost of these drugs, therapy-driven resistant virus mutations, the propensity for reinfection in cured patients, and the absence of diagnoses of most HCV carriers (3) all make the effective control of HCV infection very challenging without an effective prophylactic vaccine.

A small fraction of individuals spontaneously clear HCV infection, leading to the hypothesis that prevention of HCV infection is possible with a vaccine that can recapitulate similar immune responses (4–6). Although cellular immunity is important for the control of HCV infection, as shown in many human and chimpanzee studies (7–11), the role of neutralizing antibody in protection has also been demonstrated. Previous studies have shown a correlation between the presence of neutralizing antibodies and the clearance of acute infection (6, 12–14). Furthermore, cross-neutralizing antibodies have been shown to prevent infection or to ameliorate the course of viremia in passively immunized animals (15–18).

All successful viral vaccines developed to date have been based on the induction of neutralizing antibodies targeting the virion surface proteins (19, 20). These proteins interact with cellular receptors to mediate cell entry and to fuse with host membranes during virus uncoating (21). Broadly neutralizing antibodies targeting these proteins have been identified in natural HCV infections, although they appear slowly (22, 23). Our previous work has shown that a recombinant gpE1/gpE2 HCV vaccine is immunogenic and well tolerated in animals (24, 25) and in humans (26, 27). Vaccinated chimpanzees had a significantly reduced rate of HCV chronicity following experimental challenge, and some animals were even sterilized against homologous virus challenge (28–30). A phase I dose-ranging clinical trial has demonstrated the safety and immunogenicity of this vaccine in healthy volunteers (27). All volunteers elicited antibodies against gpE1/gpE2, and the vaccine was effective in inducing strong T helper cell responses (27). Further studies have shown that the vaccine-induced antibodies target a variety of known cross-neutralizing epitopes and that the sera of selected vaccinees inhibit *in vitro* infection by each of the seven major genotypes of HCV occurring around the world (26, 31, 32). These results demonstrated that the vaccine, although derived from a single strain, can induce very broad cross-neutralization activity. However, not all genotypes were neutralized with equal efficiencies.

HCV utilizes many strategies to evade humoral control (33). For example, in order to mask the exposure to and efficacy of neutralizing antibodies, the glycoproteins gpE1 and gpE2 are heavily glycosylated (34, 35), and the HCV virion is associated with host apolipoproteins (36–39). In addition, the virus is capable of escaping neutralization through the selection of mutants within its quasispecies population. There are several highly diverse domains identified within gpE2, including hypervariable region 1 (HVR1), HVR2, and HVR3 (the latter is also known as IgVR) (40). Of these variable domains, HVR1 has been characterized most extensively (41). This domain encompasses the first 27 amino acid (aa) residues at the amino terminus of gpE2, and evidence suggests that it is under immune-mediated selection (42). This domain is constantly evolving during chronic HCV infection (43). Interestingly, HVR1 is not critical for virus production. Virus lacking the HVR1 domain in gpE2 is infectious both *in vitro* and *in vivo* (44–47). Furthermore, cell culture-based characterization showed that HVR1-deleted virus is less dependent on one of the HCV receptors, SR-BI, for cell entry and has much higher sensitivity to various neutralizing antibodies (44, 46). More recently, Prentoe et al. reported that HVR1 shields many neutralizing epitopes and that the removal of this domain dramatically increases antibody-mediated neutralization and reduces genotype-dependent sensitivity to neutralization (48). Besides shielding many neutralizing epitopes, HVR1 also induces interfering antibodies that block adjacent access to some neutralizing antibodies (49). Taken together, these data suggested that the removal of HVR1 could enhance a HCV vaccine antigen by exposing the immune response to more-conserved regions of the glycoproteins.

Therefore, in this study, we purified recombinant gpE1/gpE2 lacking the HVR1 domain (ΔHVR1) and compared its immunogenicity with that of wild-type (WT) gpE1/gpE2. Surprisingly, we concluded that the removal of HVR1 adversely affects the immunogenicity of the glycoprotein. Although ΔHVR1 gpE1/gpE2 induced neutralizing antibodies, these antibodies had a reduced efficacy in blocking WT virus entry. These data show that ΔHVR1 gpE1/gpE2 is not a superior vaccine antigen. Combined with the demonstrated efficacy of the WT gpE1/gpE2 vaccine in the chimpanzee model (29), the present findings strongly support the further development of WT gpE1/gpE2 as a vaccine component that contains important cross-neutralizing epitopes dependent on the interaction of both gpE1 and gpE2 (50), which has been shown to be substantially more immunogenic in chimpanzees and humans than E2 alone (29).

## RESULTS

### Purification and characterization of ΔHVR1 gpE1/gpE2.

**FIG 1:**
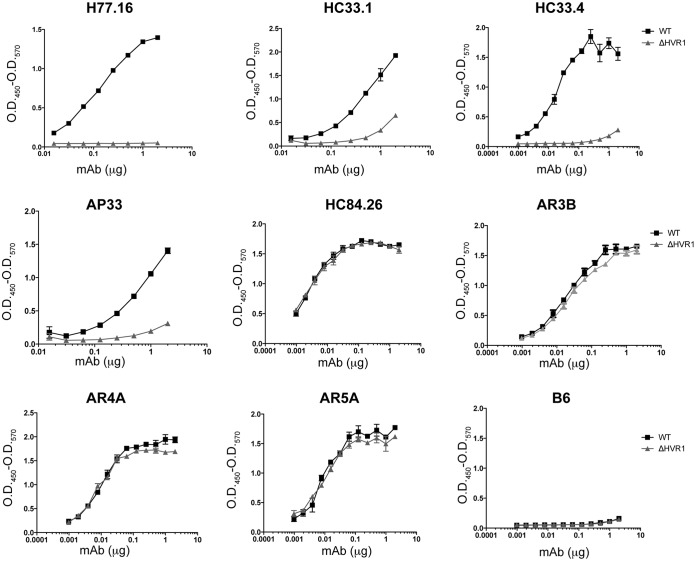
Comparison of MAb binding profiles of WT and ΔHVR1 gpE1/gpE2 antigens. Purified recombinant WT gpE1/gpE2 (WT) or gpE1/gpE2 without the HVR1 domain of gpE2 (ΔHVR1) was immobilized on ELISA plates coated with GNA-lectin. Unbound protein was removed, followed by incubation of increasing amounts of the indicated cross-neutralizing MAb. The binding of these antibodies to the recombinant protein was detected by using anti-human or anti-mouse antibody. B6 is an irrelevant human immunoglobulin control. O.D._450_, optical density at 450 nm.

We expressed recombinant ΔHVR1 gpE1/gpE2 in CHO cells as described previously for WT gpE1/gpE2 (51). In this construct, the first 27 amino acids (residues 384 to 410 using H77C polyprotein numbering) at the amino terminus of gpE2 were deleted. The isolation of gpE1/gpE2 was performed by using Galanthus nivalis agglutinin (GNA)-lectin agarose specific for mannosylated residues (30). The deletion of HVR1 did not affect the interaction of gpE2 with gpE1, and recombinant ΔHVR1 gpE1/gpE2 formed heterodimers as well as WT gpE1/gpE2 (data not shown). In order to further investigate if the deletion of HVR1 affected the folding of gpE1/gpE2, we utilized a panel of cross-reactive monoclonal antibodies (MAbs), listed in Table 1, to probe the structural differences between WT gpE1/gpE2 and ΔHVR1 gpE1/gpE2 (Fig. 1). As predicted, HVR1-specific antibody H77.16 (52) bound to WT but not to ΔHVR1 gpE1/gpE2. In contrast, gpE2-specific cross-neutralizing monoclonal antibodies AR3b and HC84.26 (53) showed no difference in binding between WT and ΔHVR1 gpE1/gpE2. Furthermore, gpE1/gpE2 heterodimer-specific cross-neutralizing antibodies AR4a and AR5a (54), which target the conformation-specific interface of gpE1 and gpE2, bound to WT and ΔHVR1 gpE1/gpE2 equally well. Interestingly, the deletion of HVR1 affected the recognition of MAbs (AP33, HC33.1, and HC33.4) (55, 56) that recognize the linear epitope I peptide region (amino acids 412 to 423) of gpE2 (57) just downstream of HVR1 (Fig. 1), implying a role for the upstream HVR1 sequence in this epitope. These results indicate that the deletion of HVR1 from gpE2 does not affect the overall folding of the gpE1/gpE2 heterodimeric complex but alters epitope I recognition.

**TABLE 1:** Monoclonal antibodies used in this study

MAb	Protein target	Conformation dependence	Critical binding residues^*a*^	Reference
H77.16	E2	No	405, 406, 408, 410	52
AP33	E2	No	413, 415, 418, 420	56
HC33.1	E2	No	413, 414, 418, 420	55
FC33.4	E2	No	408, 413, 418, 420	55
HC84.26	E2	Yes	429, 441, 442, 443, 446, 616	53
AR3b	E2	Yes	412, 416, 418, 423, 424, 523, 525, 530, 535, 540	16
AR4a	E1E2	Yes	201, 204, 205, 206, 487, 657, 658, 692, 698	54
AR5a	E1E2	Yes	201, 204, 205, 206, 639, 657, 658, 665, 692	54

a Based on H77C polyprotein numbering (residues 192 to 383 for E1 and 384 to 746 for E2). (Adapted from reference 50.)

### Immunogenicity of WT and ΔHVR1 gpE1/gpE2.

**FIG 2:**
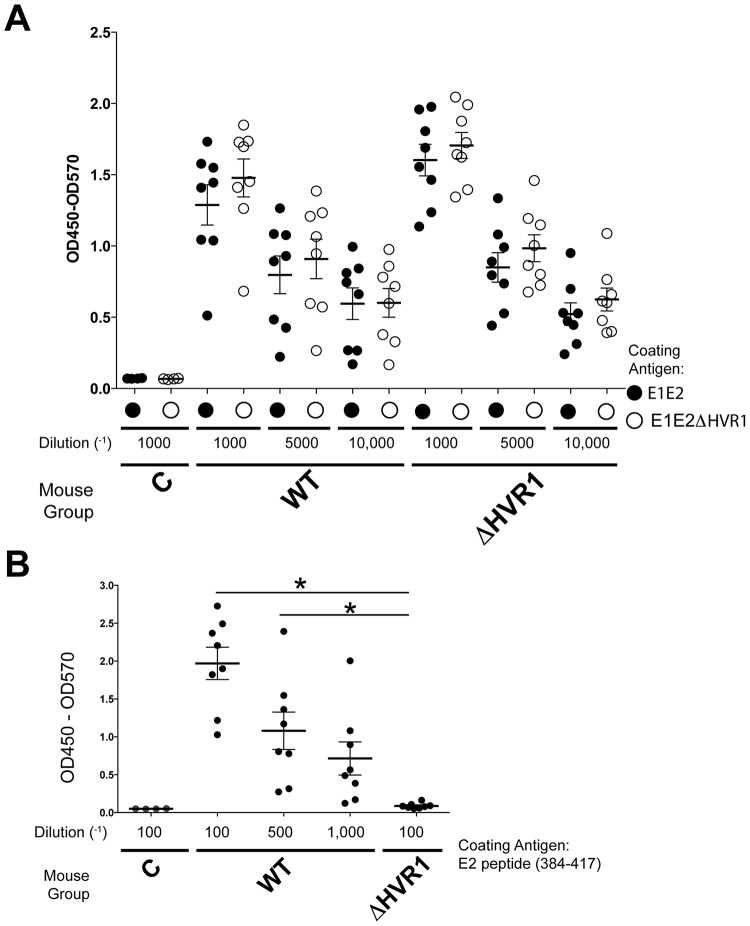
Quantitation of anti-gpE1/gpE2 (A) and antipeptide (residues 384 to 417) (B) antibodies after immunization of mice. (A) Purified recombinant gpE1/gpE2 with or without the HVR1 domain was immobilized on an ELISA plate coated with GNA. Heat-inactivated final-bleed sera from three different groups of mice were used at the indicated concentrations (control [C], gpE1/gpE2 [WT], or gpE1/gpE2 without the HVR1 domain [ΔHVR1]). Comparisons of antibody reactivity to gpE1/gpE2 with and without HVR1 at each matched concentration of sera showed no significant difference. Thus, WT and ΔHVR1 gpE1/gpE2-immunized mice showed similar titers for glycoprotein-reactive antisera. (B) An ELISA plate was coated with a peptide corresponding to residues 384 to 417 of HCV gpE2. Sera from immunized mouse groups were added at the indicated concentrations. The binding of antibody was detected with anti-mouse secondary antibody. Statistical analysis was done by one-way analysis of variance and a Tukey *post hoc* test (GraphPad); only statistically significant differences are highlighted. *, *P* < 0.05.

We first compared the immunogenicity of WT and ΔHVR1 gpE1/gpE2 antigens in mice. Figure 2A shows that immunization with the gpE1/gpE2 antigen, with or without HVR1, induced a strong antibody response to both forms of gpE1/gpE2, as measured in enzyme-linked immunosorbent assay (ELISA) formats. These elicited antibodies recognized both WT and HVR1-deleted gpE1/gpE2 proteins similarly. This suggested that the antibody response targets multiple areas of the heterodimer and is not restricted to the HVR1 domain. The titers of antibodies induced by both antigens with or without HVR1 were comparable. We also obtained similar anti-E2-specific titers between the two vaccination groups utilizing wild-type (amino acids 384 to 656) and ΔHVR1 (amino acids 412 to 656) gpE2 antigens that were isolated by a different method (58) (data not shown). For WT gpE1/gpE2 antisera, we observed antibodies that were reactive to a peptide encoding gpE2 residues 384 to 417 (Fig. 2B). At the lowest dilution (1/100) tested, antisera of ΔHVR1 gpE1/gpE2-immunized animals were not reactive to this peptide, as expected.

**FIG 3:**
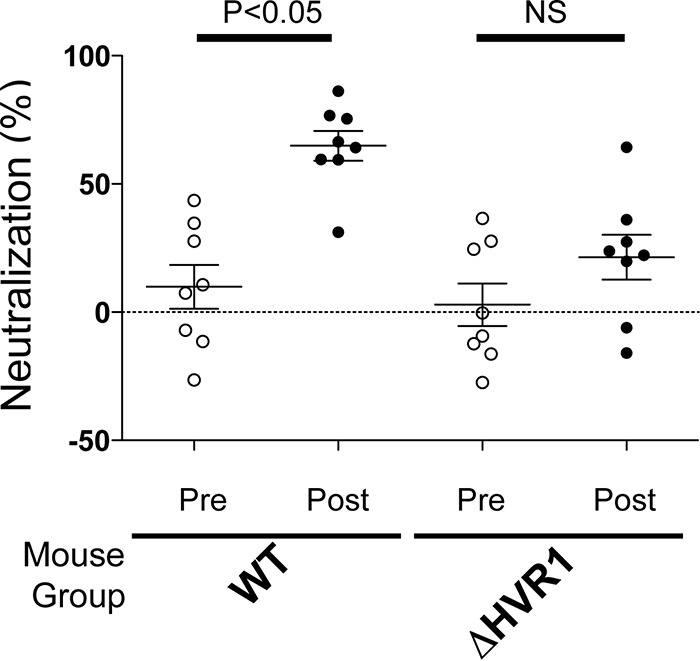
Removal of HVR1 in gpE1/gpE2 reduces neutralization activity against homologous HCVpp in vaccinated mice. Prevaccination or terminal-bleed antisera (diluted 1/100) from WT or ΔHVR1 gpE1/gpE2-vaccinated mice were examined for neutralization activity against HCVpp pseudotyped with H77C gpE1/gpE2. The amount of HCVpp entry was quantitated by measuring luciferase activity in cell extracts, as described in Materials and Methods. Neutralization activity was normalized to the luciferase activity observed in the absence of the addition of serum. Averages of data from three independent experiments performed in triplicate are shown. Statistical calculation was done by using GraphPad software. *, *P* < 0.05; NS, not significant (by one-way analysis of variance with a Tukey *post hoc* test).

**FIG 4:**
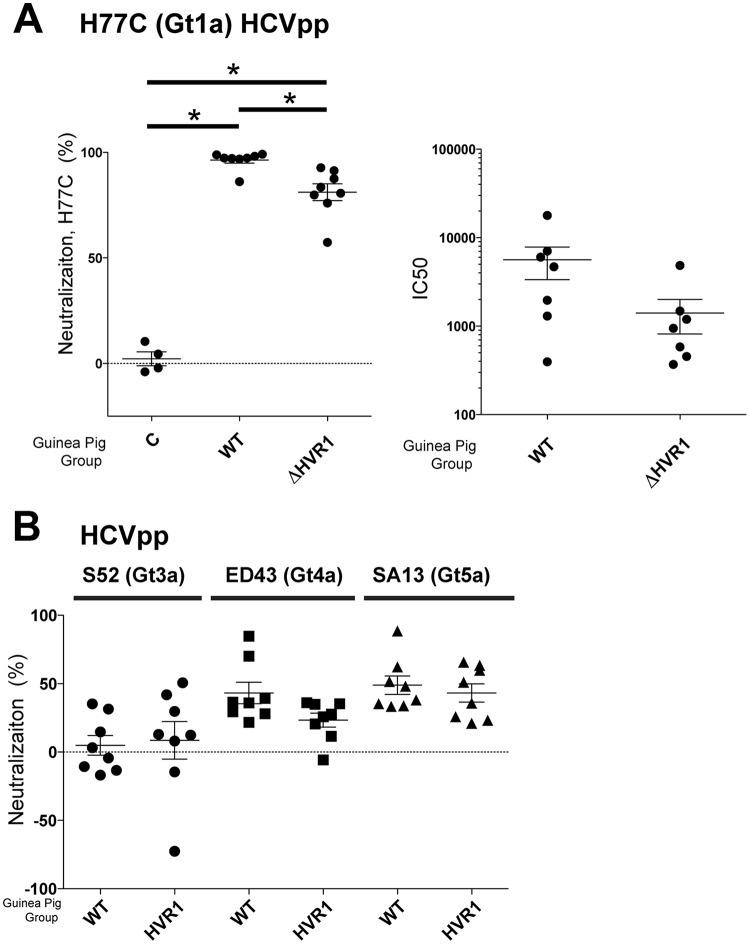
ΔHVR1 gpE1/gpE2 antigen elicits similar neutralization responses to homologous and heterologous HCVpp in vaccinated guinea pigs. Prevaccination or terminal-bleed antisera from control (C) or WT or ΔHVR1 gpE1/gpE2-vaccinated guinea pigs were examined for neutralization activity. (A) Neutralization of HCVpp pseudotyped with homologous H77C (Gt1a) gpE1/gpE2 at a 1/100 serum dilution (left) and half-maximal inhibitory concentrations (IC_50_) determined by 2-fold serial dilutions from 1/200 to 1/12,800 (right). (B) Neutralization of heterologous HCVpp pseudotyped with S52 (Gt3a), ED43 (Gt4a), or SA13 (Gt5a) gpE1/gpE2 at a 1/100 serum dilution. Neutralization activity was normalized to the luciferase level obtained by using preimmunized sera. *, *P* < 0.05 by one-way analysis of variance with a Tukey *post hoc* test. Only statistically significant differences are highlighted. Comparison of the neutralization activities of WT and ΔHVR1 gpE1/gpE2 antigens against S52/ED43/SA13 HCVpp-immunized guinea pigs showed no significant difference.

We then examined if any of the vaccine-induced antibodies could block viral infectivity. Antisera (pre- or postvaccination) from the vaccinated mice were tested for inhibition of entry using HCV-pseudotyped virus (HCVpp) comprising H77C gpE1/gpE2 (Fig. 3). We observed that ΔHVR1 gpE1/gpE2-immunized mice did not show a statistically significant increase in neutralization activity postvaccination. This was in contrast to WT gpE1/gpE2-immunized mice, which showed significant neutralizing activity against homologous HCVpp entry after vaccination. Although ΔHVR1 gpE1/gpE2 induced E2-reactive antibodies in mice (Fig. 2A), antisera from this group failed to block HCVpp entry significantly. Next, we tested the immunogenicities of WT and ΔHVR1 gpE1/gpE2 antigens in guinea pigs. In this animal model, ΔHVR1 gpE1/gpE2 induced significant neutralizing activity against homologous H77C HCVpp infection compared to the control (Fig. 4A). It appeared that the WT gpE1/gpE2-immunized guinea pigs showed higher neutralization activity than ΔHVR1 gpE1/gpE2-immunized guinea pigs at the tested dilution (Fig. 4A). The determined 50% inhibitory concentration (IC_50_) was also shown to be higher for WT gpE1/gpE2, but this was not significantly different (Fig. 4A).

**FIG 5:**
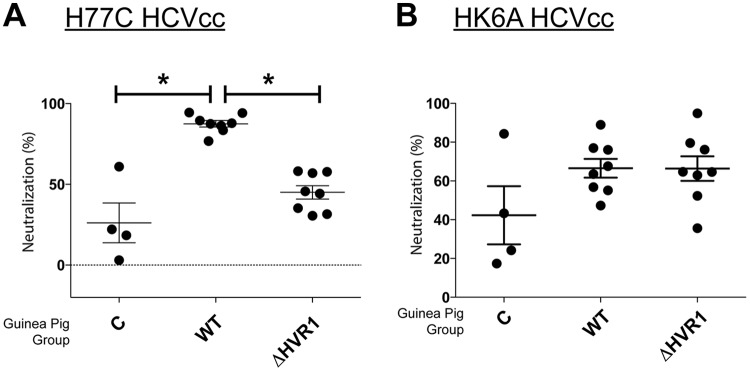
Comparison of homologous and heterologous neutralization activities of HCVcc in vaccinated guinea pigs. Prevaccination or terminal-bleed antisera (diluted 1/100) from control (C), WT, or ΔHVR1 gpE1/gpE2-vaccinated guinea pigs were examined for neutralization activity against chimeric HCVcc with structural proteins derived from H77C (Gt1a) (A) or HK6A (Gt6a) (B). Numbers of infected cells were quantitated by anti-NS5A MAb staining, and neutralization activity was normalized to the level obtained by using preimmunized sera. Averages of data from two independent experiments done in duplicate are shown. *, *P* < 0.05 by one-way analysis of variance with a Tukey posttest. Only statistically significant differences are highlighted.

Next, we examined neutralization activity against heterologous HCVpp as a test for the comparative elicitation of cross-neutralizing antibodies by the vaccine antigens. Both antigens induced similar profiles of cross-neutralizing antibodies, and efficacies were comparable between them (Fig. 4B). Cross-protection against ED43 (genotype 4a [Gt4a]) and SA13 (Gt5a) HCVpp was better than that against S52 (Gt3a) HCVpp. This is consistent with our previous findings showing that genotype 1 antigen induced weaker neutralization against genotype 2 and 3 HCVs than against genotypes 1, 4, 5, and 6 (31). Clearly, we did not observe enhanced immunogenicity and higher cross-genotype-neutralizing antibody activity after the removal of HVR1 from gpE1/gpE2. In addition, we tested homologous (Fig. 5A) and heterologous (Fig. 5B) neutralization against cell culture-derived HCV (HCVcc) and observed findings very similar to those with HCVpp. Combining data from both animal models, these results indicated that ΔHVR1 gpE1/gpE2 is not a superior antigen for inducing a protective, cross-neutralizing humoral response against HCV infection.

**FIG 6:**
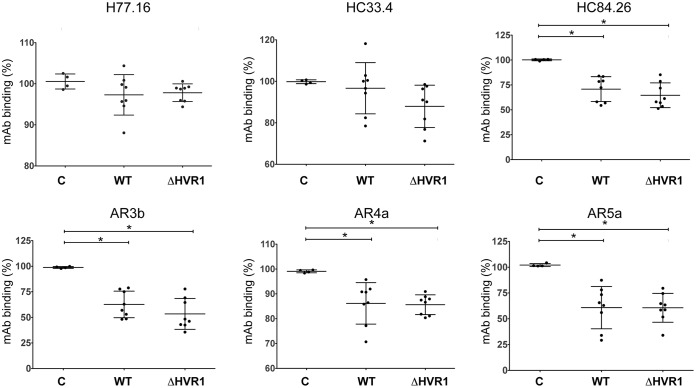
Antisera from mice vaccinated with gpE1/gpE2 compete for binding of HCV cross-neutralizing MAbs to gpE1/gpE2. Competition studies were done by using mouse antisera against a panel of cross-neutralizing monoclonal HCV antibodies. Microtiter wells coated with purified H77C gpE1/gpE2 were incubated with diluted postvaccination antiserum (1:100) in triplicate for 1 h at 37°C, followed by incubation with the indicated MAb for another hour at 37°C. The binding of the MAbs was detected with anti-human alkaline phosphatase-conjugated secondary antibodies (see Materials and Methods). For mouse MAb H77.16, the antibody was first conjugated with biotin and then detected with a streptavidin-conjugated secondary antibody. Percentages of MAb binding were calculated relative to the amount of MAb bound in the absence of mouse antiserum. Shown are mean values for each group ± ranges from two independent experiments. Vaccinated mouse groups (control [C] or vaccinated with gpE1/gpE2 [WT] or HVR1-deleted gpE1/gpE2 [ΔHVR1]); E2-specific antibodies H77.16, HC33.4, HC84.26, and AR3b; and gpE1/gpE2-specific antibodies AR4a and AR5a were used (Table 1). Statistical analysis was done by one-way analysis of variance with a Tukey *post hoc* test (GraphPad); only statistically significant difference are highlighted. *, *P* < 0.05. Nonsignificant differences are not labeled.

We further investigated the mechanism of reduced virus neutralization from antisera of ΔHVR1 gpE1/gpE2-immunized mice. Previously, we showed that WT gpE1/gpE2 induced antibodies that target different conserved neutralizing epitopes (50, 51). Therefore, we performed competition ELISAs and assessed the ability of the vaccinated mouse antisera to block the binding of various cross-neutralizing antibodies that target the envelope glycoproteins. We used MAbs H77.16, HC33.4, HC84.26, AR3b, AR4a, and AR5a in this study to encompass a variety of important neutralization epitopes (Table 1). The profiles of competition assays between the sera derived from WT and ΔHVR1 gpE1/gpE2-vaccinated mice showed very similar patterns (Fig. 6). We observed that both WT and ΔHVR1 antisera competed similarly for the binding of HC84.26, AR3b, AR4a, and AR5a antibodies to gpE1/gpE2. These data suggested that both antigens induced antibodies targeting similar arrays of neutralizing epitopes. In contrast, both the HVR1-specific H77.16 antibody and the HC33.4 antibody (targeting epitope I located immediately downstream of HVR1) were not significantly competed by antisera from either vaccination group (Fig. 6).

### Testing neutralization against HCVpp pseudotyped with ΔHVR1 gpE1/gpE2.

**FIG 7:**
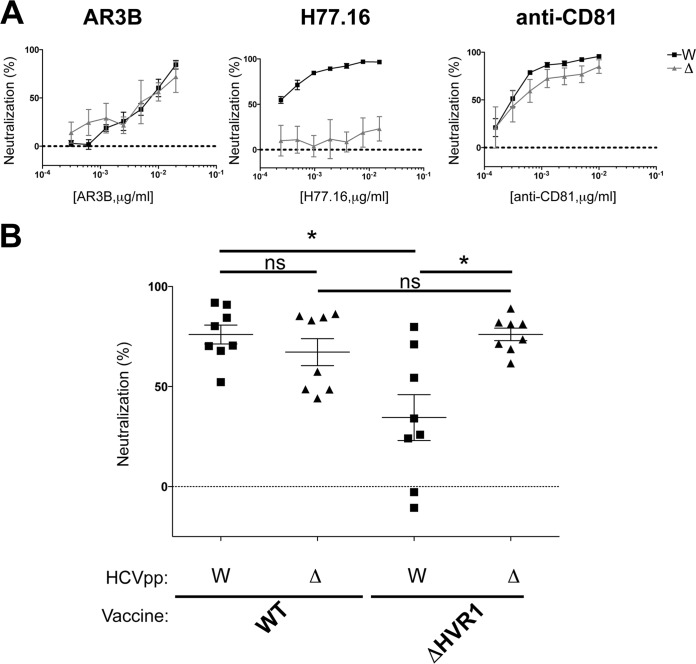
Comparison of neutralization responses to WT and ΔHVR1 HCVpp (H77C) in vaccinated mice. (A) Increasing concentrations of specific monoclonal antibody AR3b or H77.16 or anti-CD81 were examined for neutralization of HCVpp pseudotyped with either WT gpE1/gpE2 (W) or gpE1/gpE2 lacking the HVR1 domain (Δ). In order to support HCVpp assembly, gpE2 in ΔHVR1 HCVpp encoded two adaptive mutations, H261R and Q444R, as described previously (46). (B) Terminal antisera (diluted 1/100) from mice immunized with either WT or ΔHVR1 gpE1/gpE2 antigens were examined for neutralization activity. Neutralization activities of postimmunization sera were normalized to those of preimmunized sera. *, *P* < 0.05; ns, nonsignificant (by one-way analysis of variance with a Tukey *post hoc* test [GraphPad]).

In order to explore further if the lack of an HVR1-specific antibody response played a role in the reduced neutralization observed for ΔHVR1 gpE1/gpE2-immunized mice, we compared neutralization activities against HCVpp pseudotyped with either WT or ΔHVR1 gpE1/gpE2 (Fig. 7). HCVpp pseudotyped with ΔHVR1 gpE1/gpE2 was described previously (46). Two compensatory mutations in gpE1 and gpE2 (H261R and Q444R) were needed in order to restore entry to the wild-type level (46). Interestingly, while the virus lacking HVR1 of gpE2 showed hypersensitivity to many broadly neutralizing monoclonal antibodies (48), HCVpp with or without HVR1 showed a neutralization sensitivity similar to that of cross-neutralizing monoclonal antibody AR3b (Fig. 7A, left). As expected, we showed that HCVpp devoid of HVR1 is resistant to neutralization by H77.16, an antibody targeting HVR1 (Fig. 7A, middle). The HVR1-specific H77.16 neutralizing antibody was capable of neutralizing only WT and not ΔHVR1 HCVpp. Both WT and ΔHVR1 HCVpp are affected by antibodies targeting the host receptor CD81, although it appeared that ΔHVR1 HCVpp is less affected (Fig. 7A, right).

We observed that antisera after ΔHVR1 gpE1/gpE2 vaccination showed a reduced efficiency in neutralizing WT HCVpp (Fig. 7B). This is consistent with the data shown in Fig. 3. However, a similar neutralization of HCVpp pseudotyped with ΔHVR1 gpE1/gpE2 was observed by using antisera from vaccination with either WT or HVR1-deleted gpE1/gpE2 antigens (Fig. 7B), indicating that both antigens elicit similar levels of neutralizing antibodies targeting epitopes other than HVR1. The presence of HVR1 in the vaccine antigen leads to an enhancement of the neutralization of the homologous virus, presumably due to the effect of neutralizing antibodies targeting HVR1.

## DISCUSSION

HCV employs multiple strategies to evade the host immune response. HVR1 plays a critical role in this escape, as evidenced by its continuous change under selection from HVR1-targeted neutralizing antibodies (42, 43, 59). Based on the recent findings that HVR1-deleted HCVs exhibited hypersensitivity to neutralizing antibodies and reduced genotype-specific variation in their neutralization (44, 45, 48, 60), it became relevant to compare the immunogenicities of WT and ΔHVR1 gpE1/gpE2 antigens as vaccine candidates. While we observed that both antigens induced strong gpE1/gpE2-reactive antibodies in ELISAs (Fig. 2A), ΔHVR1 gpE1/gpE2 vaccine antisera showed reduced neutralization against homologous H77C HCVcc and HCVpp in both mice and guinea pigs (Fig. 3, 4A, and 5A), even though there was no detectable difference in our competitive ELISAs (Fig. 6). Presumably, since WT gpE1/gpE2 elicited a strong antibody response to a peptide comprising aa 384 to 417 (Fig. 2B), in addition to raising neutralizing antibodies against conserved epitopes targeted by MAbs HC84.26, AR3A, AR4A, and AR5A (Fig. 6), WT gpE1/gpE2 must in addition elicit neutralizing antibodies targeting aa 384 to 417 other than those targeted by MAbs H77.16 and HC33.4 and/or elicit neutralizing antibodies to epitopes comprising in part this peptide region. In guinea pigs, levels of neutralization against HCVpp or HCVcc of heterologous genotypes (genotypes 3, 4, 5, and 6) were lower than that against homologous H77C HCVpp but comparable between the WT and ΔHVR1 gpE1/gpE2 vaccine groups (Fig. 4B and 5B). Our data indicate that the deletion of the HVR1 domain from recombinant gpE1/gpE2 offers no advantages in the induction of cross-neutralizing antibodies and decreases the neutralization of homologous virus.

In this study, we observed that WT and ΔHVR1 gpE1/gpE2 antigens had nearly identical binding profiles for several cross-neutralizing HCV MAbs, with the exception of the epitope I-specific MAbs AP33, HC33.1, and HC33.4 (Fig. 1). This was an unexpected finding based on studies that reported an enhanced sensitivity of ΔHVR1 HCVcc to patient-derived and HCV-neutralizing MAbs (44, 45, 48, 60). It is possible that structural differences between recombinant gpE1/gpE2 and assembled viral particles, such as the higher-order assembly of gpE1/gpE2 and/or the association with lipoproteins, may account for these differences. Consistent with our antigen characterization, both WT and ΔHVR1 gpE1/gpE2-vaccinated mice induced very similar anti-gpE1/gpE2 titers (Fig. 2), and antisera competed with various cross-neutralizing HCV MAbs similarly (Fig. 6). In addition, our analyses using WT and ΔHVR1 gpE1/gpE2 antigens indicated that in both vaccine groups, the majority of anti-E2 antibodies targeted regions outside the HVR1 domain (Fig. 2).

Antisera from ΔHVR1 gpE1/gpE2-vaccinated animals had reduced neutralizing activity against homologous H77 HCVpp, relative to antisera from WT gpE1/gpE2-vaccinated animals, and this result was more pronounced in mice than in guinea pigs (Fig. 3, 4A, and 5A). Higher variability in virus neutralization was observed for mice than for guinea pigs in a previous study (24) and likely reflects intrinsic differences between the animal models. Antisera from guinea pigs of both vaccination groups showed similar neutralization activities against heterologous HCVpp or HCVcc (Fig. 4B and 5B). This shows that the removal of HVR1 does not enhance the induction of cross-neutralizing antibodies. Consistent with this finding, our results showed that antisera from both WT and ΔHVR1 gpE1/gpE2-vaccinated animals can neutralize HCVpp pseudotyped with ΔHVR1 HCVpp similarly well (Fig. 7B). Collectively, our observations indicate that (i) recombinant ΔHVR1 gpE1/gpE2 does not adopt a conformation that significantly enhances the exposure of cross-neutralizing epitopes and (ii) immunization with ΔHVR1 gpE1/gpE2 does not generate superior neutralization responses from vaccinated animals compared to WT gpE1/gpE2.

To date, recombinant WT gpE1/gpE2 remains the only HCV vaccine candidate with proven prophylactic efficacy at reducing the HCV carriage rate in vaccinated chimpanzees (or in any animal model [29]). WT gpE1/gpE2 has been shown to be significantly more immunogenic in chimpanzees than gpE2 alone (29) and contains important discontinuous cross-neutralizing epitopes requiring the interaction of both envelope glycoproteins (54). We have shown that this antigen is well tolerated and capable of eliciting broad cross-neutralizing antibodies in humans as well as strong lymphoproliferative responses (31). In addition, cellular immune responses to HCV nonstructural proteins are associated with the eradication of viremia in humans (61, 62), and both CD4^+^ and CD8^+^ T cells have been shown to be required for the eradication of viremia in chimpanzees (10, 11). Previously, we showed that an adjuvanted HCV nonstructural polyprotein (lacking gpE1/gpE2) was capable of eliciting broad virus-specific CD4^+^ and CD8^+^ T cells that ameliorated acute HCV infection and acute hepatitis following experimental challenge (29), as did a vaccine based on the use of replication-defective viral vectors to deliver HCV nonstructural genes (63). It may be appropriate, therefore, to elicit both arms of the adaptive immune response in an optimal HCV vaccine formulation. Recently, we developed an expression-and-purification process to enable the scale-up and delivery of our HCV vaccine to the human population (51). We intend to initiate clinical testing of WT gpE1/gpE2 with and without HCV nonstructural protein antigens in the near future.

In summary, our data show that the removal of gpE2 HVR1 results in a loss of reactivity with a neutralizing MAb targeting this linear region (H77.16) and a large reduction in reactivity with cross-neutralizing MAbs targeting the linear epitope I region (57) immediately downstream (AP33, HC33.1, and HC33.4) (Fig. 1). In contrast, highly cross-neutralizing MAbs targeting various conformational epitopes within gpE2 and gpE1/gpE2 bind identically to WT and ΔHVR1 gpE1/gpE2 antigens (Fig. 1). Clearly, there was no increase in immunoreactivity observed using ΔHVR1 gpE1/gpE2 against any cross-neutralizing MAb. Antisera from mice vaccinated with either antigen competed similarly with cross-neutralizing MAbs targeting conformational epitopes within gpE2 and gpE1/gpE2 (Fig. 6). While antisera from mice vaccinated with WT gpE1/gpE2 neutralized homologous H77 HCVpp, antisera from mice vaccinated with ΔHVR1 gpE1/gpE2 showed markedly reduced neutralization (Fig. 3), as did antisera from guinea pigs (Fig. 4), indicating the importance of the involvement of additional neutralizing epitopes in the neutralization of homologous virus. When tested against many diverse genotypes using either HCVpp or HCVcc, it was clear that ΔHVR1 gpE1/gpE2 does not improve the neutralization of WT viruses compared with the WT gpE1/gpE2 vaccine (Fig. 4 and 5). However, the ΔHVR1 gpE1/gpE2 vaccine neutralizes homologous ΔHVR1 HCVpp better than WT HCVpp (Fig. 7), perhaps reflecting differences in the cell entry process as a result of altering the interaction between HVR1 and the virus entry receptor SR-BI (46, 64). In conclusion, deleting HVR1 from the recombinant E1E2 vaccine candidate offers no advantages in neutralizing homologous or heterologous viruses.

## MATERIALS AND METHODS

### Cell cultures and antibodies.

CHO cells stably expressing recombinant gpE1/gpE2 constructs derived from the genotype 1a H77C strain (GenBank accession number AF009606) with either WT gpE2 (amino acids 384 to 746) or gpE2 without HVR1 (amino acids 412 to 746) were propagated in Iscove's modified Dulbecco's medium (Thermo Fisher Scientific, Waltham, MA, USA) containing 10% heat-inactivated fetal bovine serum (FBS) (Thermo Fisher Scientific), 0.1 mM sodium hypoxanthine–0.016 mM thymidine (HT supplement; Thermo Fisher Scientific), 0.002 mM methotrexate, 100 U/ml penicillin, and 100 μg/ml streptomycin (PenStrep; Invitrogen, Carlsbad, CA, USA). Huh7.5 cells were propagated in Dulbecco's modified Eagle's medium (Thermo Fisher Scientific) containing 10% heat-inactivated FBS (Omega Scientific, Tarzana, CA, USA), 0.1 mM nonessential amino acids (Invitrogen, Carlsbad, CA, USA), and penicillin and streptomycin (PenStrep; Invitrogen). The mouse MAb anti-cluster of differentiation 81 (CD81) clone JS-81 (BD Biosciences, Franklin Lakes, NJ, USA), mouse isotype control IgG1 (R&D Systems, Minneapolis, MN, USA), anti-HCV MAbs (H77.16, AP33, HCV33.1, HC33.4, HC84.26, AR3b, AR4a, and AR5a), and human anti-HIV antibody B6 were described previously (16, 53–56). Anti-HCV MAbs and B6 were kindly provided by Steven Foung (Stanford University), Mansun Law (The Scripps Research Institute), and Arvind Patel (University of Glasgow).

### Expression and purification of recombinant gpE1/gpE2 antigens.

The expression and purification of recombinant gpE1/gpE2 proteins were described previously (30, 51). The WT gpE1/gpE2 glycoprotein coding region from H77C (genotype 1a) (GenBank accession number AF009606) (amino acids 192 to 746) or the coding region of gpE1/gpE2 without HVR1 (deletion of amino acids 384 to 411), each of which was preceded by the signal peptide sequence for tissue plasminogen activator (tPA), was inserted into the SpeI/MluI site of the pTRIP lentiviral vector bearing an internal ribosome entry site (IRES)-Aequorea coerulescens green fluorescent protein (ACGFP) reporter (65). Lentiviral particles were generated in HEK-293T cells according to a previously reported method (65), and CHO cells were transduced with packaged lentivirus. Green fluorescent protein (GFP)-positive CHO cells expressing WT or ΔHVR1 gpE1/gpE2 were sorted by flow cytometry using a BD FACSAria III cell sorter (BD Biosciences), and the suspension was then adapted in Procho4 medium (Lonza, Walkersville, MD, USA) with 6% FBS in 250-ml shaker flasks (Corning, Corning, NY, USA). Cells were expanded in 3-liter spinner flasks (Corning), and recombinant gpE1/gpE2 was purified from CHO cell extracts by using GNA-lectin agarose (Vector Laboratories, Burlingame, CA, USA), as reported previously (30, 51). The GNA eluate fraction was loaded onto a hydroxyapatite (HAP) column (catalog number 158-8000; Bio-Rad, Hercules, CA, USA), and the flowthrough was concentrated with a 50,000-molecular-weight-cutoff centrifugal filter unit (EMD Millipore, Billerica, MA, USA). The final antigen reached at least 90% purity.

### Immunization of animals and collection of serum samples.

Female CB6F1 mice (Charles River Laboratories, Montreal, QC, Canada) (5 to 7 weeks old) or Hartley guinea pigs (Medimabs, Montreal, QC, Canada) used for immunization were cared for in accordance with Canadian Council on Animal Care guidelines. Experimental methods were reviewed and approved by the University of Alberta Health Sciences Animal Welfare Committee. Recombinant WT or ΔHVR1 gpE1/gpE2 antigens (2 μg [mouse] or 7.5 μg [guinea pig]) were mixed at a 1:1 ratio in a volume with 75 μg alum and 7.5 μg monophosphoryl lipid A (MPLA) (Vaccigrade; InvivoGen, San Diego, CA, USA). Mice were given intramuscular injections (35-μl final injection volume) at days 0, 14, 28, and 56. Guinea pigs received subcutaneous injections (100-μl final injection volume) at days 0, 14, 42, and 90. Prevaccination blood samples were collected at day 0, and postvaccination blood samples (terminal bleeds) were obtained 14 days after the final immunization. Whole-blood samples were centrifuged at 5,000 × g for 15 min, and sera were collected and heat inactivated at 56°C for 30 min. Serum samples were stored in aliquots at −80°C until use.

### ELISA. (i) gpE1/gpE2 ELISA.

Microtiter plate wells (Corning) were coated with 1 μg GNA-lectin (Sigma-Aldrich, St. Louis, MO, USA) in phosphate-buffered saline (PBS) overnight at 4°C and then blocked for 1 h with 4% bovine serum albumin (BSA) (Sigma-Aldrich) in PBS containing 0.2% Tween 20 (PBST). After washing of the wells with PBST, WT or ΔHVR1 gpE1/gpE2 antigens (100 ng/well) were added for 1 h. gpE2-specific MAbs (H77.16, AP33, HC33.1, HC33.4, HC84.26, and AR3b) (16, 52, 53, 55, 56), gpE1/gpE2-specific MAbs (AR4a and AR5a) (54), or a control MAb (B6) (16) was added for 1 h (50 μl/well) and detected by an anti-human or anti-mouse horseradish peroxidase-conjugated secondary antibody (1:10,000; Jackson Immuno Research, West Grove, PA, USA) and KPL peroxidase substrate (SeraCare Life Sciences, Milford, MA). The absorbance (450 to 570 nm) was read by using an Enspire plate reader (Perkin-Elmer, Waltham, MA, USA).

### (ii) gpE1/gpE2 and peptide ELISAs.

Microtiter plates were coated with H77C gpE1/gpE2 or gpE1/ΔHVR1-gpE2 (amino acids 412 to 656) overnight at 4°C in PBS. For peptide ELISAs, wells coated with an N-terminal biotinylated peptide corresponding to H77C residues 387 to 417 (biotin-CETHVTGGNAGRTTAGLVGLLTPGAKQNIQLINTN; GLBiochem, Shanghai, China) at 2 μg/well were blocked with 4% BSA in PBS for 1 h. Sera from vaccinated mice were diluted in PBST and added to the plates for 1 h (50 μl/well). gpE1/gpE2 and peptide-specific antibodies were detected by using a horseradish peroxidase-conjugated goat anti-mouse secondary antibody (1:10,000; Cedarlane Laboratories, Burlington, ON, Canada) and KPL peroxidase substrate (SeraCare Life Sciences, Milford, MA). The absorbance was read at 450 to 570 nm as described above. Absorbance values from three independent experiments are expressed as means ± standard errors of the means (SEM).

### (iii) Competition ELISA.

Mouse sera (terminal bleeds) were assessed for competition with conformation-specific HCV MAbs for gpE1/gpE2 binding based on a method described previously (50). Briefly, 96-well microtiter plates were coated with GNA-HAP-purified WT gpE1/gpE2 H77C in carbonate coating buffer overnight at 4°C and blocked with 1% casein (Sigma-Aldrich) in PBS–0.5% (vol/vol) Tween 20. Diluted mouse sera were incubated for 1 h in wells coated with gpE1/gpE2. HCV-specific (H77.16 [biotin linked], HC33.4, HC84.26, AR3b, AR4a, and AR5a) or control (mouse IgG1 [mIgG1] isotype control [biotin linked] and B6 anti-HIV human IgG1 [huIgG1] isotype control) MAbs were then added for 1 h at a subsaturating concentration normally resulting in 70% maximal binding. Bound HCV-specific MAbs was detected by an anti-human alkaline phosphatase (AP)-conjugated secondary antibody (1:10,000; Jackson ImmunoResearch) and a *p*-nitrophenyl phosphate (Sigma-Aldrich) substrate. For mouse monoclonal antibody H77.16, the antibody was first biotinylated by using the EZ-link NHS-PEG4 biotinylation kit (Thermo Fisher Scientific), and binding was detected by using AP-conjugated streptavidin (1:4,000; Sigma-Aldrich), as described previously (50). The absorbance was read at 405 to 495 nm as described above. Values were calculated as a percentage of MAb binding relative to the MAb bound in the absence of serum. Data are plotted as means ± SEM from two independent experiments.

### Production of HCV-pseudotyped virus (HCVpp) and cell culture-derived HCV (HCVcc) and neutralization assays.

Plasmids encoding chimeric HCV genomes representing H77C and HK6A were described previously (66). DNA templates were generated by linearizing plasmids using XbaI, and infectious RNAs were generated by using a T7 RiboMAX large-scale RNA production system (Promega, Madison, WI). RNA was subsequently purified by using the RNeasy minikit (Qiagen, Hilden, Germany).

HCVcc was produced by using a previously described protocol (67). Cells were washed twice with ice-cold PBS and subsequently resuspended to 1.5 × 10^7^ cells/ml. Four hundred microliters of the cell suspension was mixed with 5 μg *in vitro*-transcribed RNA encoding the HCV genome in 2-mm-gap electroporation cuvettes. Five pulses of 860 V (99 μs with 1.1-s intervals) were delivered by using the ECM 830 ElectroSquare porator (BTX, Holliston, MA). Postelectroporation, cells were incubated at room temperature for 10 min before plating. Precleared medium was collected as virus stocks at either day 3 or 4 postelectroporation. The virus titer (50% tissue culture infectious dose [TCID_50_]) was determined by limited dilution as described previously (67). HCVpp expressing a luciferase reporter were generated as described previously (68). HCVpp pseudotyped with ΔHVR1 gpE1/gpE2 was described previously and encodes two compensatory mutations, H261R and Q444R (46). For neutralization assays, human hepatoma cells (Huh7.5) were plated onto polylysine-coated 96-well plates 1 day prior to infection. HCVcc or HCVpp was diluted and premixed with heat-inactivated diluted sera for 1 h at 37°C, followed by addition to Huh7.5 cells. At 6 h postinfection, the antibody-virus inoculum was replaced with fresh culture medium. Cells were either processed at 48 h postinfection using the Bright-glo luciferase assay system (Promega, Madison, WI, USA) for HCVpp or fixed with methanol for HCVcc. Luminescence (HCVpp) was measured by using an Enspire plate reader (Perkin-Elmer). Infection (HCVcc) was detected by using a mouse anti-NS5a antibody (9e10), as described previously (67). Neutralization activity normalized to that of prevaccination sera was calculated by using the formula % neutralization = (pre − post)/pre × 100, where pre/post represents the luciferase activity or number of infected cells detected after incubation with either the pre- or postvaccination sera. Alternatively, neutralization activity was calculated by normalization with the luciferase activity of HCVpp without incubation of any sera.
